# Dielectric Properties of BaTiO_3_–Epoxy Nanocomposites in the Microwave Regime

**DOI:** 10.3390/polym13091391

**Published:** 2021-04-25

**Authors:** Hsin-Yu Yao, Yi-Wen Lin, Tsun-Hsu Chang

**Affiliations:** Department of Physics, National Tsing Hua University, 101, Section 2, Kuang Fu Road, Hsinchu 300044, Taiwan; s5te633v@hotmail.com (H.-Y.Y.); dk394xup643@gmail.com (Y.-W.L.)

**Keywords:** high-*k* epoxy nanocomposites, microwave dielectric characterization, effective medium theory, Bragg reflector, antireflection coating

## Abstract

We synthesized BaTiO_3_–epoxy nanocomposites (particle size < 100 nm) with volume fractions up to 25 vol. %, whose high-frequency complex permittivity was characterized from 8.2 to 12.5 GHz. The maximum dielectric constant approaches 9.499 with an acceptable loss tangent of 0.113. The dielectric loss gradually saturates when the particle concentration is higher than 15 vol. %. This special feature is an important key to realizing high-*k* and low-loss nanocomposites. By comparing the theoretical predictions and the experimental data, four applicable effective-medium models are suggested. The retrieved dielectric constant (loss tangent) of 100-nm BaTiO_3_ nanopowder is in the range of 50–90 (0.1–0.15) at 8.2–12.5 GHz, exhibiting weak frequency dispersion. Two multilayer microwave devices—total reflection and antireflection coatings—are designed based on the fabricated nanocomposites. Both devices show good performance and allow broadband operation.

## 1. Introduction

In the era of fifth-generation mobile networks, plenty of works have been devoted to developing integrable microwave components, such as microstrip antenna [1], circulators [2], capacitors [3,4,5], filters [6], low-loss waveguides [7], resonators [8] and multi-functional surface coatings [9,10]. To improve their electrical performance, miniaturization of device size and suppression of material loss are essential. One crucial key for reaching these goals is to increase the material dielectric constant while keeping its dielectric loss low. High-*k* composites composed of polymer host and nanosized dopants are of great interest due to their promising capability for enhancing the dielectric constant with good mechanical processability (low cost and low processing temperature) and high electrical/optical/thermal/mechanical tunability. 

Many different types of composites have been reported. Polymers doped with conductive fillers, such as graphene [11], silver [12] or other metals [13], are able to increase the dielectric constant drastically, but at the expense of low compatibility with printed circuit boards (owing to large leakage current) and high signal attenuation (due to severe Ohmic loss). On the other hand, ferromagnetic dopants, e.g., Fe_3_O_4_ [14], and ferroelectric dopants such as PZT [15], TiO_2_ [16,17,18], SrTiO_3_ [19], BaTiO_3_ [3,4,5,20,21,22,23,24,25,26,27,28,29,30,31,32] or other oxides [17,18], are also good candidates. BaTiO_3_–polymer composites have attracted great attention among various combinations due to their high-*k* properties with high mechanical tunability and thermal stability. These features facilitate applications in energy storage [3,4,5], electromechanical transducers [20,21], capacitive sensors [22] or optical devices with tunable index [23]. Furthermore, BaTiO_3_–epoxy (BTEP) composites have been intensively studied and regarded as a potential solution to meet the demand for low loss [31,32]. Kuo et al. [3,4] fabricated samples through different sintering processes to investigate the effect of heating temperature. For the 30 vol. % BTEP composite, the dielectric constant can be arbitrarily controlled between 15 and 30 at 100 kHz. To further increase the available doping fraction, Cho et al. [27] synthesized a series of BTEP composites with maximum particle concentration achieving 60 vol. %, whose dielectric constant approaches 60 at 100 kHz. In addition, the dependence of the dielectric constant on the particle size was analyzed: the larger the particle size, the larger the dielectric constant. A similar trend was observed by Dang et al. [28], where the volume fraction of the BTEP composite was up to 70 vol. %, and a broadband dielectric characterization was conducted in the kHz–MHz region. Recently, Phan et al. [25] demonstrated that the dielectric constant of a nanocomposite could be significantly enhanced when a silane coupling agent was used for surface modification. A 5 wt. % BTEP composite can exhibit a dielectric constant of around 7. It should be noted that those previous studies only focused on the low-frequency band (<1 GHz).

Regarding the high-frequency dielectric characterization, Yang et al. [30] discovered that the dielectric constants of 40 vol. % BTEP samples at 3–18 GHz were, respectively, 11 and 15 when 100 nm and 200 nm nanoparticles were doped. The corresponding dielectric loss tangents range between 0.04 and 0.08. Similar high-frequency data were reported in Ref. [31], where the dielectric constant of 20 vol. % BTEP composite is around 7 at 4–6 GHz. Recently, Zhang et al. [32] suggested that the dielectric loss can be reduced by constructing a proper buffer layer at the dopant–host interface. A maximum dielectric constant of 7.5–8.5 with a low loss tangent of 0.02–0.03 for a 20 vol. % BTEP nanocomposite was demonstrated at 8.2–12.5 GHz. Although a high-*k* and low-loss BTEP nanocomposite has been achieved, there is still no complete investigation of the relationship between the BaTiO_3_ concentration and the resulting composite’s electric performance at the high-frequency region (>1 GHz). In order to predict the effective permittivity of the mixture, many effective-medium theories (EMTs) have been developed over the past century (see Section 3.3). However, several unsolved problems still remain. Firstly, since there is no direct method to measure the dielectric constant of powders [27], the complex permittivity of nanosized BaTiO_3_ particles (<100 nm) at the GHz-THz regime is still unknown. Secondly, most of the EMTs were developed under different assumptions; therefore, the question of which models are applicable for the fabricated samples should be carefully considered. Thirdly, up to now, whether these EMTs can predict the imaginary (loss) part of composite remains questionable [31,32].

In this study, a series of BaTiO_3_–epoxy nanocomposites (particle size < 100 nm) with different doping volume fractions up to 25% were synthesized. The morphonology of samples was characterized by scanning electron microscopy (SEM), and their high-frequency dielectric properties were characterized at 8.2–12.5 GHz. Ten famous effective-medium theories were thoroughly reviewed and examined by our experimental data. The best-fitting effective-medium models will be suggested, which can retrieve the dielectric properties of BaTiO_3_ nanopowder at the high-frequency region and the system’s morphological factor. With such information, two proof-of-principle microwave devices are proposed: a total reflection coating and an antireflection coating. Both designs show good performance and allow broadband operation.

## 2. Materials and Methods

**Figure 1:**
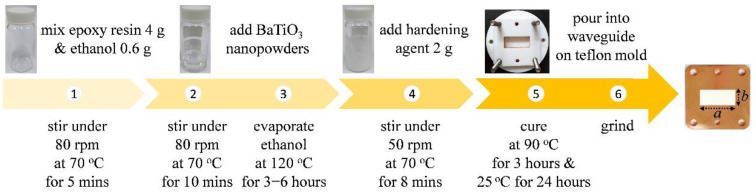
Flow chart for sample preparation. Detailed processes are described in the main text.

The commercially available epoxy resin was supplied by HOMYTECH Co., Ltd., Taoyuan, Taiwan (product #: DTE-206LA/LB), and the nanosized BaTiO_3_ powders with an average diameter below 100 nm were purchased from Nanomaterial, Inc., Houston, TX, USA (product #: US3835). The powders were coated with polyvinylpyrrolidone (PVP) as the surfactant for good dispersion in ethanol. The densities of epoxy resin and BaTiO_3_ powders were 1.13 and 5.85 g/cm^3^, respectively. Five BTEP nanocomposites with different volume fractions (5, 10, 15, 20 and 25 vol. %) were synthesized based on the flow chart illustrated in Figure 1. Firstly, 4.0 g epoxy resin and 0.6 g ethanol solvent (>99.5 vol. %) were homogeneously mixed and heated up to 70 °C for 5 min (step 1 in Figure 1). The BaTiO_3_ powders with proper weight (calculated according to the desired volume fraction and the densities) were added into the epoxy–ethanol solution. The mixture was stirred under 80 rpm for 10 min to form a homogeneous and stable solution without aggregation or precipitation (step 2). Then, the sample was heated up to 120 °C for 3–6 h to evaporate ethanol completely (step 3). After removing the solvent, the solution was cooled down to 70 °C, and then 2 g hardening agent (100 (epoxy):50 (hardener) by weight) was added. The mixture was slowly stirred under 50 rpm for 8 min to distribute the agent thoroughly (step 4). Relatively low stirring speed was used so as to avoid the entrapment of microbubbles. Before the final curing process, the mixture was degassed and poured into an X-band waveguide (width (a) × height (b) × thickness (L) is 22.86 mm × 10.16 mm × 2.98 mm). It should be noted that the waveguide was made of oxygen-free copper and mounted on a Teflon mold for easily demolding after curing. The curing process was conducted in a rotary heater under 90 °C for 3 h, followed by room temperature for 24 h (step 5). Finally, the demolded waveguide sample was ground (finely up to 3000 meshes, step 6). The fabrication processes of composites with different volume fractions were similar.

## 3. Results and Discussion

### 3.1. Microscale Morphologies of BaTiO_3_–Epoxy Nanocomposites

**Figure 2:**
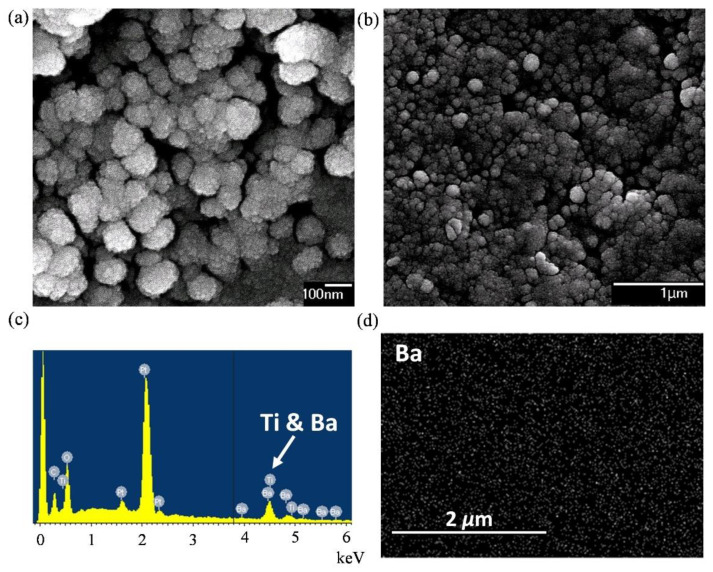
SEM images of (**a**) pure BaTiO_3_ nanopowders and (**b**) 25 vol. % BaTiO_3_–epoxy nanocomposite. (**c**) EDS of 25 vol. % sample and (**d**) the corresponding Barium distribution using EDS mapping.

Figure 2a shows the SEM image of pure BaTiO_3_ nanopowders. The observed particle sizes range between 60 and 130 nm with an average value of 95 nm. This is slightly larger than the specification provided by the vendor (50 nm). We observe that the particle shape is close to ellipsoid rather than sphere, which will be further discussed in Section 3.3. Figure 2b displays the SEM image of the 25 vol. % BTEP nanocomposite. The images of other samples are similar. It can be seen that BaTiO_3_ particles with sizes around 100 nm were homogeneously dispersed within the epoxy matrix. No apparent aggregation was observed. Figure 2c,d are the energy-dispersive X-ray spectra (EDS) of the 25 vol. % sample and the corresponding EDS mapping of barium. The peak at around 4.5 keV in Figure 2c indicates the existence of barium and titanium. Figure 2d confirms the homogeneous distribution of BaTiO_3_ nanopowders in the epoxy matrix.

### 3.2. High-Frequency Complex Permittivity of BaTiO_3_–Epoxy Nanocomposites

**Figure 3:**
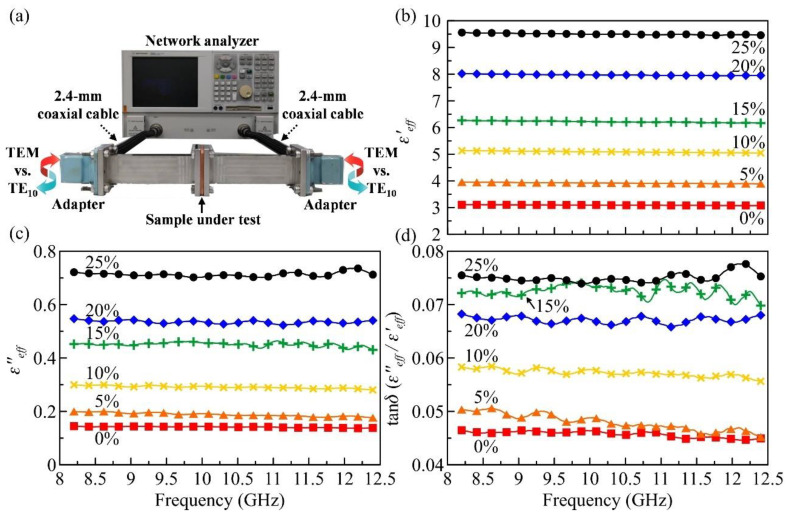
(**a**) Experimental setup for microwave permittivity characterization. Frequency dependence of (**b**) ${\varepsilon^{\prime }}_{eff}$, (**c**) ${\varepsilon^{\prime \prime }}_{eff}$ and (**d**) tanδ of BaTiO_3_–epoxy nanocomposites with six different volume fractions.

The complex permittivity of BTEP nanocomposites was characterized at the X band (8.2–12.5 GHz). Figure 3a shows a photograph of the experimental setup. A performance network analyzer (Agilent E8363B, Agilent Technologies, Santa Clara, CA, USA) was connected to two X-band adapters by 2.4 mm coaxial cables. These two adapters convert the TEM mode from the coaxial cables to the TE_10_ mode in the rectangular waveguides. Note that the adapters with waveguide extensions can be calibrated in advance by the standard open-short-load kit. The well-calibrated ends were attached to the waveguide sample to measure the two-port scattering matrix. Since the system is symmetric and reciprocal, we expect $S_{11}=S_{22}$ and $S_{12}=S_{21}$, which can be respectively expressed as

(1) $$S_{11}=r+\frac{tt^{\prime }r^{\prime }\exp\left(2ik_{z}L\right)}{1-{r^{\prime }}^{2}\exp\left(2ik_{z}L\right)},$$

(2) $$S_{21}=\frac{tt^{\prime }\exp\left(ik_{z}L\right)}{1-{r^{\prime }}^{2}\exp\left(2ik_{z}L\right)},$$

where $r=-r^{\prime }=(k_{z0}-k_{z})/(k_{z0}+k_{z})$, $t=2k_{z0}/(k_{z0}+k_{z})$ and $t^{\prime }=2k_{z}/(k_{z0}+k_{z})$, representing the Fresnel interface scattering coefficients under external (without prime) and internal (with prime) excitations. $k_{z0}=\sqrt{\omega^{2}-\omega_{c}^{2}}/c$ and $k_{z}=\sqrt{\varepsilon_{eff}\omega^{2}-\omega_{c}^{2}}/c$ respectively correspond to the propagation constants in the empty and the loaded waveguides, in which ω is the frequency of electromagnetic wave, c is the speed of light in vacuum, and $\omega_{c}=c\pi /a$ is the cutoff frequency of TE_10_ mode. The complex permittivity of the sample under test ($\varepsilon_{eff}={\varepsilon^{\prime }}_{eff}+i{\varepsilon^{\prime \prime }}_{eff}$) can be retrieved from the measured scattering data by the Nicolson–Ross–Weir method [33].

Figure 3b,c demonstrate the retrieved ${\varepsilon^{\prime }}_{eff}$ and ${\varepsilon^{\prime \prime }}_{eff}$ of six samples, respectively. For the pure epoxy (0 vol. %), its ${\varepsilon^{\prime }}_{eff}$ (${\varepsilon^{\prime \prime }}_{eff}$) is around 3.092 (0.141), with negligible frequency dispersion. With the increase in particle volume fraction, both ${\varepsilon^{\prime }}_{eff}$ and ${\varepsilon^{\prime \prime }}_{eff}$ significantly increase due to the participation of high-index BaTiO_3_ powders [3,4,27,28,29,30,31,32] and the interfacial mismatching between the dopant and the host [32]. As the volume fraction approaches 25%, the average dielectric constant (${\varepsilon^{\prime }}_{eff}$) reaches 9.499. Similar to the pure epoxy, ${\varepsilon^{\prime }}_{eff}$ of the composites exhibit weak frequency dispersion over the measured spectral window.

Regarding the loss tangent ($\tan\delta \equiv {\varepsilon^{\prime \prime }}_{eff}/{\varepsilon^{\prime }}_{eff}$) in Figure 3d, there exist small wiggles that become relatively obvious at high volume fraction (>15%). This phenomenon might result from random internal scattering between the defects inside the high-concentration samples. Moreover, we observed that the increment of the loss tangent becomes saturated after 15 vol. %. The loss tangent of the 15 vol. % nanocomposite reaches 0.073, higher than that of the 10 vol. % sample (0.057) by 0.016. However, tanδ starts to fluctuate when the doping ratio increases from 15 to 25 vol. %. At 25 vol. %, tanδ slightly increases to 0.075, very close to the measured value of 15 vol. %. The loss saturation implies that a low-loss and ultrahigh-*k* compound can be achieved if the concentration of BaTiO_3_ is further increased. This perspective is supported by the data in Ref. [30]. The reported loss tangent of the 40 vol. % BTEP sample ranges between 0.06 and 0.07 at 10 GHz, very close to the measured value of our 25 vol. % composite (0.075). However, these observations seem to contradict the data published in the recent work of Zhang et al. [32], in which the loss tangent linearly increases with the doping ratio till 20 vol. %, no matter what surfactants were coated. The dependence of dielectric loss on the BaTiO_3_ fraction and the origin of loss saturation deserve more in-depth studies.

### 3.3. Revisit of Effective Medium Theories and Dielectric Properties of BaTiO_3_ Nanopowders 

Numerous theories are able to predict the effective permittivity of composites ($\varepsilon_{eff}$) and explain the underlying physics. In the following, we enumerate ten representative models. Only one kind of particle with permittivity $\varepsilon_{p}$ is considered, which is assumed to be homogeneously distributed in a host matrix with permittivity $\varepsilon_{h}$. The volume fraction of the particle (host) is denoted as $v_{p}$ ($v_{h}=1-v_{p}$). Lichtenecker’s law of mixing [34,35,36,37] has been recognized as a general approach with broad applicability:

(3) $$\varepsilon_{eff}^{n}=v_{p}\varepsilon_{p}^{n}+v_{h}\varepsilon_{h}^{n},$$

in which the power n is related to the system’s microgeometry (topology). The value of n lies in between ±1, corresponding to Wiener upper and lower limits [38,39]. n=1/2 corresponds to the straightforward linear mixing of complex refractive indices (CRI), while n=1/3 is the so-called Landau–Lifshitz–Looyenga (LLL) model [40]. The LLL model is derived by using the Taylor expansion and integral method [41] to describe the successive inclusion of an infinitesimal amount of particles. Since there is no specific assumption on the particle shape, it is applicable to the mixtures, including irregularly shaped dopants, such as the porous materials [17,18].

On the other hand, Lichtenecker’s logarithmic mixing law (abbreviated as Log) can be deduced from the differential form of Equation (3) [36,42] by substituting n=0:

(4) $$\varepsilon_{eff}=v_{p}\log\varepsilon_{p}+v_{h}\log\varepsilon_{h},$$

It describes a system exhibiting two well-dispersed phases without connected layers in parallel or series [36]. Empirically, Equations (3) and (4) are valid for the composites with middle volume fractions. From the experiment conducted by Cho et al. [27], Equation (4) can work well even for the heavily doped case, with $v_{p}$ achieving 60%. However, both Equations (3) and (4) can be applied only if the difference between $\varepsilon_{p}$ and $\varepsilon_{h}$ is small because of the employment of Taylor approximation.

The Maxwell–Garnet (MG) model [43], also known as the Maxwell–Wagner–Sillars approach [27,43,44,45,46,47,48], has been developed by the consideration of spherical inclusions ($\varepsilon_{p}$) embedded in a homogeneous background with fixed permittivity ($\varepsilon_{h}$). After analyzing the effective polarizability of a dielectric sphere and generalizing it to the multiple-particle case by the Clausius-Mossotti format [49], $\varepsilon_{eff}$ satisfies

(5) $$\frac{\varepsilon_{eff}-\varepsilon_{h}}{\varepsilon_{eff}+2\varepsilon_{h}}=v_{p}\frac{\varepsilon_{p}-\varepsilon_{h}}{\varepsilon_{p}+2\varepsilon_{h}},$$

or an alternative form of

(6) $$\varepsilon_{eff}=\varepsilon_{h}\frac{2\varepsilon_{h}+\varepsilon_{p}+2v_{p}\left(\varepsilon_{p}-\varepsilon_{h}\right)}{2\varepsilon_{h}+\varepsilon_{p}-v_{p}\left(\varepsilon_{p}-\varepsilon_{h}\right)}.$$

To relax the limitation on the particle shape, Yamada, Ueda and Kitayama (YUK) [15] modified the MG format by introducing a morphological factor N, which is dependent on the shape of the dopant. For ellipsoidal inclusion with transversal symmetry, Equation (6) could be transformed into

(7) $$\varepsilon_{eff}=\varepsilon_{h}\frac{\left(1-N\right)\varepsilon_{h}+N\varepsilon_{p}+\left(1-N\right)v_{p}\left(\varepsilon_{p}-\varepsilon_{h}\right)}{\left(1-N\right)\varepsilon_{h}+N\varepsilon_{p}-Nv_{p}\left(\varepsilon_{p}-\varepsilon_{h}\right)},$$

where N is defined as:

(8) $$N=N_{x}=\frac{x_{0}y_{0}z_{0}}{2}\int_{0}^{\infty }\frac{du}{(x_{0}^{2}+u)\sqrt{\left(x_{0}^{2}+u)(y_{0}^{2}+u)(z_{0}^{2}+u\right)}},$$

and $x_{0}$, $y_{0}$ and $z_{0}$ are the ellipsoidal axis lengths of the dopant. Note that $N_{x}=$ 1/3, 1 and 0 for sphere, disc and rod, respectively [17,18]. In the case of the sphere (N= 1/3), Equation (7) reduces to Equation (5). 

If the dopant concentration is high or the permittivity mismatching between the dopant and the host is severe, the effective background permittivity should significantly deviate from $\varepsilon_{h}$ [43]. As a consequence, the MG model is limited, applicable for low-concentration and low-contrast cases only. To address this problem, Bruggeman [50] suggested replacing the host’s permittivity $\varepsilon_{h}$ by the permittivity of the effective medium $\varepsilon_{eff}$ during the derivation of Equation (5) [41,51,52,53,54]. This results in a refreshed form:

$$\frac{\varepsilon_{eff}-\varepsilon_{h}}{3\varepsilon_{eff}}=v_{p}\frac{\varepsilon_{p}-\varepsilon_{h}}{\varepsilon_{p}+2\varepsilon_{eff}}.$$

The above expression can be alternatively written as

(9) $$v_{p}\frac{\varepsilon_{p}-\varepsilon_{eff}}{\varepsilon_{p}+2\varepsilon_{eff}}+v_{h}\frac{\varepsilon_{h}-\varepsilon_{eff}}{\varepsilon_{h}+2\varepsilon_{eff}}=0,$$

which is easier to generalize for the composites with multiple constituents. Equation (9) is a quadratic function of $\varepsilon_{eff}$, indicating

(10) $$\varepsilon_{eff}=\frac{1}{4}\left\{\left(2-3v_{p}\right)\varepsilon_{h}-\left(1-3v_{p}\right)\varepsilon_{p}+\sqrt{{\left[\left(2-3v_{p}\right)\varepsilon_{h}-\left(1-3v_{p}\right)\varepsilon_{p}\right]}^{2}+8\varepsilon_{p}\varepsilon_{h}}\right\}.$$

It is the so-called symmetric Bruggeman formulae (sBM) [31,52], capable of estimating the composite’s permittivity with high particle concentration.

To further remove the constraint on the particle shape, Polder and van Santen (PvS) modified Equation (9) based on the aforementioned depolarization factor [55], yielding

(11) $$\varepsilon_{eff}=\varepsilon_{h}\frac{1}{1-\frac{1}{3}v_{p}\left(\varepsilon_{p}-\varepsilon_{h}\right)\sum_{s=x,y,z}\frac{1}{\varepsilon_{eff}+\left(\varepsilon_{p}-\varepsilon_{eff}\right)N_{s}}},$$

where $N_{s}$ can be calculated from Equation (8) by generalizing the first $\left(x_{0}^{2}+u\right)$ to $\left(s_{0}^{2}+u\right)$ with s=x, y, or z. It is not surprising that Equation (11) equals Equation (10) when $N_{x}=N_{y}=N_{z}=$1/3 for spherical inclusions. Although the PvS model has supplemented the Maxwell–Garnet model, it is applicable for the composites with low permittivity contrast [17,18].

To extend the model for the high-contrast cases, Bruggeman started from the differential form of Equation (5) and assumed that the MG theory is valid for an infinitesimal increment in the particle’s volume fraction and permittivity [50]. After integrating the particle fraction from 0 to $v_{p}$ corresponding to the composite permittivity varying from $\varepsilon_{h}$ to $\varepsilon_{eff}$ [41], one could obtain

(12) $$1-v_{p}=\frac{\varepsilon_{p}-\varepsilon_{eff}}{\varepsilon_{p}-\varepsilon_{h}}\sqrt[{3}]{\frac{\varepsilon_{h}}{\varepsilon_{eff}}}$$

Equation (12) is the primary form of differential effective-medium theory (DEM) for spheres, whose generalizations for randomly oriented anisotropic inclusions were reviewed in Ref. [41]. Recently, Schller et al. [17,18] derived a general form:

(13) $$1-v_{p}=\frac{\varepsilon_{p}-\varepsilon_{eff}}{\varepsilon_{p}-\varepsilon_{h}}{\left(\frac{\varepsilon_{h}}{\varepsilon_{eff}}\right)}^{\left(\frac{-3N^{2}+3N}{3N+1}\right)}{\left[\frac{\left(1+3N\right)\varepsilon_{p}+\left(5-3N\right)\varepsilon_{h}}{\left(1+3N\right)\varepsilon_{p}+\left(5-3N\right)\varepsilon_{eff}}\right]}^{\left(\frac{12N-18N^{2}-2}{9N^{2}-12N-5}\right)},$$

where N is the depolarization factor (Equation (6)). Equation (13) (denoted as MS-DEM) is valid for ellipsoidal dopants with transversal symmetry, combining the advantages of the PvS model (Equation (11), valid for high concentration and ellipsoidal inclusions) and DEM (Equation (12), valid for high concentration and high permittivity contrast). Note that for sphere (N= 1/3), Equation (13) is equivalent to Equation (12).

In line with this, Jayasunder and Smith (JS) considered the interactions between neighboring spheres [56] and modified the mean fields inside the constituting materials used in Kerner format [57]. This results in

(14) $$\varepsilon_{eff}=\frac{v_{h}\varepsilon_{h}+v_{p}\varepsilon_{p}\left[\frac{3\varepsilon_{h}}{\left(\varepsilon_{p}+2\varepsilon_{h}\right)}\right]\times \left[1+\frac{3v_{p}\left(\varepsilon_{p}-\varepsilon_{h}\right)}{\left(\varepsilon_{p}+2\varepsilon_{h}\right)}\right]}{v_{h}+v_{p}\left[\frac{3\varepsilon_{h}}{\left(\varepsilon_{p}+2\varepsilon_{h}\right)}\right]\times \left[1+\frac{3v_{p}\left(\varepsilon_{p}-\varepsilon_{h}\right)}{\left(\varepsilon_{p}+2\varepsilon_{h}\right)}\right]}.$$

The term in the first bracket derived by Böttcher [44,45,46,54] corresponds to the mean-field ratio between the particle and the host. The second bracket manifests the particle–particle interaction effect. In the absence of the second bracket, Equation (14) reduces back to Equation (5). Since the interaction has been considered, this method is claimed to be valid for a large volume fraction and high permittivity contrast. However, Equation (14) was derived for spheres, and thus it might lack the applicability for anisotropic inclusions. Table 1 briefly summarizes the pros and cons of these ten effective-medium models.

**Figure 4:**
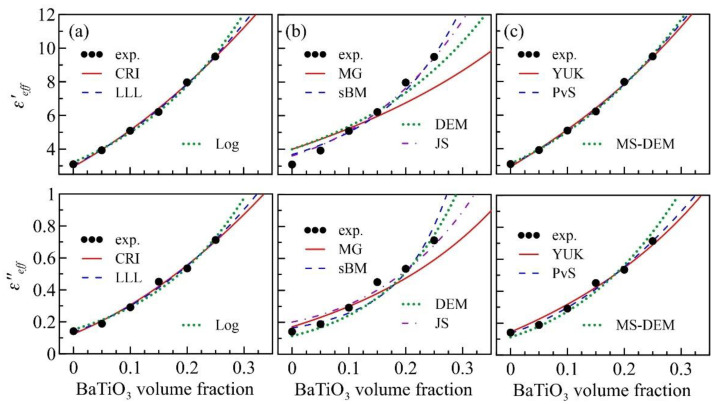
Comparison of the experimental results and the predictions from (**a**) CRI, LLL and Log; (**b**) MG, sBM, DEM and JS; and (**c**) YUK, PvS and MS-DEM. The top (bottom) panel shows ${\varepsilon^{\prime }}_{eff}$ (${\varepsilon^{\prime \prime }}_{eff}$ ) versus volume fraction.

In the following, these models are fitted to the experimental data to retrieve the complex permittivity of epoxy ($\varepsilon_{h}={\varepsilon^{\prime }}_{h}+i{\varepsilon^{\prime \prime }}_{h}$) and BaTiO_3_ nanoparticle ($\varepsilon_{p}={\varepsilon^{\prime }}_{p}+i{\varepsilon^{\prime \prime }}_{p}$). For the models that allow ellipsoidal inclusions (i.e., YUK, PvS, and MS-DEM), the depolarization factor N will be retrieved, too. The results are listed in Table 2. The values in the parentheses are the errors of $\varepsilon_{h}$ as compared to the measured epoxy permittivity. The last two columns are the sums of all deviation squares defined by $\Delta^{\prime }={\sum \left({\varepsilon^{\prime }}_{eff,\mathrm{EMT}}-{\varepsilon^{\prime }}_{eff,\exp.}\right)}^{2}$ and $\Delta^{\prime \prime }={\sum \left({\varepsilon^{\prime \prime }}_{eff,\mathrm{EMT}}-{\varepsilon^{\prime \prime }}_{eff,\exp.}\right)}^{2}$. For discussion, the ten models are classified into three groups—I. Lichtenecker’s law of mixing (CRI, LLL and Log), II. EMTs with sphere inclusions (MG, sBM, DEM and JS) and III. EMTs with ellipsoidal inclusions (YUK, PvS and MS-DEM), the fitting results of which are displayed in Figure 4a,b,c, respectively.

The predictions from the Lichtenecker mixing laws (Figure 4a) are in good agreement with the experimental data (with small $\Delta^{\prime }\sim$0.07 and $\Delta^{\prime \prime }\sim$0.003) and the retrieved host permittivity ${\varepsilon^{\prime }}_{h}$ and ${\varepsilon^{\prime \prime }}_{h}$ fairly match the measured ones, with errors below 5% and 15%, respectively. On the contrary, Figure 4b reveals that the MG, sBM, DEM and JS models fail to fit the data (with large $\Delta^{\prime }>$1 and $\Delta^{\prime \prime }>0.1$). More than 20% deviation between the measured and the retrieved $\varepsilon_{h}$ was observed. The bad fitting result implies that the present composites might exhibit anisotropic microstructures. Once the particle shape limitation is relaxed, the theories’ validity can be significantly improved, as shown in Figure 4c. For the PvS and MS-DEM models, the total deviation $\Delta^{\prime }$ is below 0.05, and the retrieved ${\varepsilon^{\prime }}_{h}$ is very accurate, with an error below 1%. As expected, the predictions from these two models are very close because the maximum concentration is only 25 vol. %, such that the particle–particle interaction is insignificant.

Although most models in groups I and III seem applicable, two of them—Log and YUK—still exhibit relatively large deviations, with $\Delta^{\prime }\sim$0.1, leading to unreasonably large ${\varepsilon^{\prime }}_{p}$ (>200) and ${\varepsilon^{\prime \prime }}_{p}$ (>50). Thus, these two models will be excluded from the following discussion. In summary, the remaining four effective-medium models—CRI, LLL, PvS and MS-DEM—are applicable to our nanocomposites with middle volume fraction (< 30%) and middle permittivity contrast (< 100). The retrieved particle dielectric constant (${\varepsilon^{\prime }}_{p}$) lies between 50 and 90 (average: 66.594), and the particle loss tangent (${\varepsilon^{\prime \prime }}_{p}/{\varepsilon^{\prime }}_{p}$) is 0.113. Reference [25] reported a similar dielectric constant of 62.3 at 10 kHz for a 93 nm BaTiO_3_ nanoparticle. It implies that the frequency dispersion of the dielectric constant of BaTiO_3_ nanoparticles is weak, below 10 GHz. According to the N retrieved by PvS and MS-DEM (< 0.1), the shape of BaTiO_3_ particles tends to be rod-like rather than sphere-like. This result matches the SEM images provided in Figure 2a,b. Note that the retrieved particle permittivity is consistent with the trend reported by Ref. [27], as the particle size is extended to below 100 nm.

### 3.4. Microwave Applications of High-k Nanocomposites: Total Reflection Coating and Antireflection Coating 

**Figure 5:**
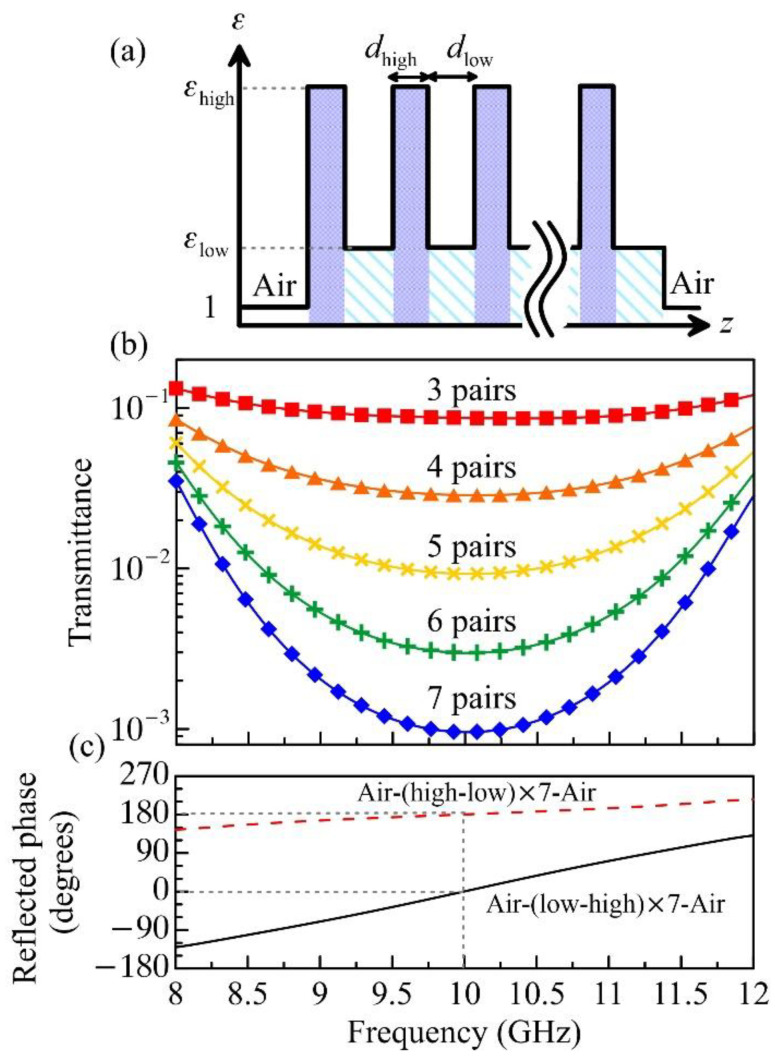
(**a**) Permittivity profile of Bragg total reflection coating. (**b**)Transmittance of Bragg coating made of alternating 25 vol. % BTEP nanocomposite and pure epoxy layers. The simulation results are plotted in symbols. (**c**) Reflected phases of the Bragg coatings with high–low–high (red dashes) and low–high–low (black) layer orders.

The high-*k* and low-loss BaTiO_3_–epoxy nanocomposites have great potential applications in microwave and THz-wave technology [31,32]. In the following, two proof-of-concept designs, namely a total reflection coating and antireflection coating, will be proposed and verified by a 3D full-wave simulator (high-frequency structure simulator (HFSS), ANSYS, Canonsburg, PA, USA). To reflect an EM wave, metals usually serve as the best candidate at a low-frequency regime. However, Ohmic loss at high frequency, e.g., the THz band, will become unacceptably high. For low-loss operation, all-dielectric mirrors were invented, such as photonic metasurface [58,59], gratings [60,61] or Bragg reflectors [7,8,62]. Bragg reflectors consist of alternating layers with high ($\varepsilon_{\mathrm{high}}$) and low ($\varepsilon_{\mathrm{low}}$) permittivity, whose thicknesses are respectively denoted as $d_{\mathrm{high}}$ and $d_{\mathrm{low}}$ (see Figure 5a). Since strong roundtrip destructive interference is required to suppress transmission, the quarter-wavelength stack condition should be satisfied, yielding dhigh=λ0/4Re[εhigh] and dlow=λ0/4Re[εlow], where $\lambda_{0}$ denotes the center operating wavelength [63]. For further enhancing the termination and broadening the bandwidth, the contrast between $\varepsilon_{\mathrm{high}}$ and $\varepsilon_{\mathrm{low}}$ needs to be as high as possible [63], such that the strong reflection at the layer–layer interface ensures a robust photonic bandgap.

To fulfill these guidelines, we respectively choose the 25 vol. % nanocomposite (highest concentration) and the pure epoxy (lowest concentration) as the high-index and low-index layers, implying $\varepsilon_{\mathrm{high}}=$9.499 + *i* × 0.713, $\varepsilon_{\mathrm{low}}=$3.092x + *i* × 0.141, $d_{\mathrm{high}}=$ 2.43 mm and $d_{\mathrm{low}}=$ 4.26 mm (for bandgap center at 10 GHz). Under free-space surface-normal incidence, the overall scattering can be calculated by the transfer matrix method [7,63]. Figure 5b displays the transmittance of the designed Bragg mirrors with various numbers of Bragg pairs (i.e., a set of 25 and 0 vol. %). The agreement between the theory (curves) and the HFSS simulation (symbols) is excellent. The more Bragg pairs, the stronger the photonic bandgap, leading to strong suppression of minimum transmission from −10 dB (3 pairs) to −30 dB (7 pairs). The average transmittance for the 7-pair mirror is below −20 dB, with high reflectance of around 90% at the X band. It is worth noting that the reflected phase at the bandgap center can be designed at 180° or 0° by switching the sequence of layers from high–low–high to low–high–low configuration (see Figure 5c). However, most common metals can provide higher reflection with broader bandwidth at microwave, whose reflected phase is fixed at 180°. High reflection with zero reflected phase change implies that the surface field on the Bragg reflector can be enhanced, facilitating more interesting applications in reflective-type microwave surface spectroscopy, microscopy and thin-film characterization [9].

**Figure 6:**
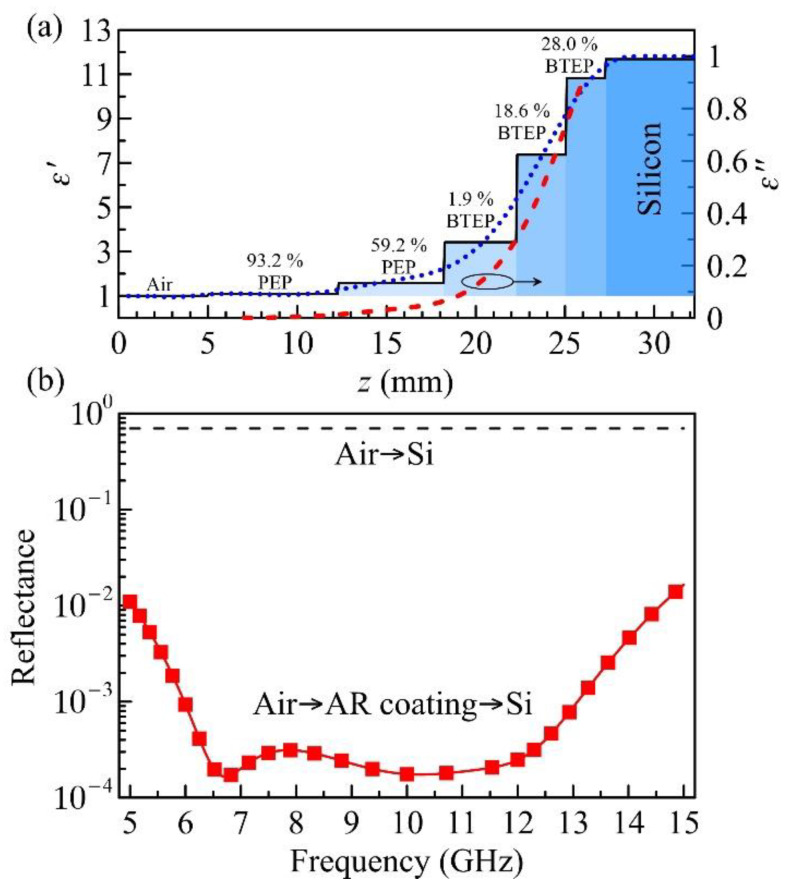
(**a**) Permittivity profile of the antireflection coating. The doping volume fraction and composite type of each layer are indicated. The continuous complex permittivity profile (blue dots for $\varepsilon^{\prime }$ and red dashed for $\varepsilon^{\prime \prime }$) is the fitting result of a 10-order polynomial. (**b**) Reflectance of silicon substrate coated with and without antireflection coating (red squared and black dashes, respectively). Symbols are the HFSS simulation results.

In contrast to the total reflection, the antireflection coating is able to suppress the reflection for energy saving [64]. The multilayer antireflection coating is known for its capability in ultra-broadband operation. Yao et al. recently proposed a new multilayer design based on silicon–HDPE nanocomposites for matching widely used silicon in free space. Since the dopant and the matching target have the same permittivity (11.683), several matching layers require unrealistic particle doping concentrations (>70 vol. %). In this investigation, we propose to replace the Si–HDPE layers with BaTiO_3_–epoxy nanocomposites. Owing to the high dielectric constant of BaTiO_3_ (${\varepsilon^{\prime }}_{p}>$50)**,** the required maximum volume fraction can be less than 30%, which is much easier to achieve. A five-layer antireflection coating is designed by the binominal matching theory [9,65], whose permittivity profile is illustrated in Figure 6a, and the detailed doping parameters are listed in Table 3.

Among these five layers, the first two layers with the required dielectric constants lower than that of the pure epoxy (3.092) can be realized by highly porous epoxy (PEP). Recent research has confirmed that low-*k* epoxy with porosity higher than 90% can be synthesized [66]. On the other hand, high-*k* layers are realized by the BTEP nanocomposites with a maximum volume fraction of less than 30%. Table 3 summarizes the coating parameters, in which the required volume fractions are calculated by the LLL (MS-DEM) model for PEP-type (BTEP-type) composites. Figure 6b demonstrates the reflectance from Si with or without antireflection coating calculated by TTM (curves) and HFSS (symbols) at 5–15 GHz. We assume that the permittivity of every layer is nearly dispersion-less in this frequency range. It is clear that the designed coating can reduce the reflection by more than two orders of magnitude. The available fractional bandwidth for −20 dB reflection reaches 100%, representing a promising feature of the multilayer antireflection coating.

## 4. Conclusions

In summary, BaTiO_3_–epoxy nanocomposites (dopant size < 100 nm) have been successfully fabricated with concentrations up to 25 vol. %. Their high-frequency dielectric properties were characterized from 8.2 to 12.5 GHz. The results show that the maximum dielectric constant achieves 9.499 with an acceptable loss tangent of 0.075. Saturation of dielectric loss with the increment of doping volume fraction (> 15 vol. %) indicates the possibility of ultrahigh-*k* and low-loss nanocomposites. The applicability of the ten effective-medium models was reviewed. Based on these theories, we simultaneously retrieved the complex permittivity of epoxy matrix and BaTiO_3_ nanofiller. By comparing the retrieved permittivity of the matrix (0 vol. %) with the measured data, four best-fitting models are suggested, i.e., CRI, LLL, PvS and MS-DEM. The retrieved dielectric constant (loss tangent) of the 100-nm BaTiO_3_ nanoparticle is 66.594 (0.113) at 10 GHz. To the best of our knowledge, this is the first time that the high-frequency complex permittivity of BaTiO_3_ nanoparticles is reported. It is of great importance for predicting the dielectric properties of BTEP nanocomposites with high concentrations. Finally, two microwave devices composed of the BTEP nanocomposites are proposed: the total reflection coating and the antireflection coating. The transmittance of the seven-pair reflection coating is reduced to below −20 dB throughout nearly the entire X band (8.2–12.5 GHz). On the other hand, the five-layer antireflection coating can suppress the reflectance of the air–silicon interface to below −20 dB from 5–15 GHz, corresponding to 100% fractional bandwidth. 

## Figures and Tables

**Table 1:** Scope of applicability of the ten effective-medium theories.

Model	Volume Fraction	Permittivity Contrast	Particle Shape
CRI (Equation (3), *n* = 1)	low	low	irregular
LLL (Equation (3), *n* = 1/3)	middle	low	irregular
Log (Equation (4))	middle	low	irregular
MG (Equation (6))	low	low	spherical
YUK (Equation (7))	middle	middle	ellipsoidal
sBM (Equation (9))	high	low	spherical
PvS (Equation (11))	high	low	ellipsoidal
DEM (Equation (12))	high	high	spherical
MS-DEM (Equation (13))	high	high	ellipsoidal
JS (Equation (14))	high	high	spherical

**Table 2:** Complex permittivity of BaTiO_3_ powder $\varepsilon_{p}$ and epoxy $\varepsilon_{h}$ and depolarization factor (N ) retrieved by the ten EMTs.

Model	${\varepsilon^{\prime }}_{p}$	${\varepsilon^{\prime \prime }}_{p}$	${\varepsilon^{\prime }}_{h}$	${\varepsilon^{\prime \prime }}_{h}$	N	$\Delta^{\prime }$	$\Delta^{\prime \prime }$
CRI	51.109	5.005	2.952 (−4.54%)	0.125 (−11.92%)		0.075	0.002
LLL	70.657	7.582	3.040 (−1.70%)	0.134 (−5.03%)		0.043	0.002
Log	261.021	42.002	3.212 (+3.86%)	0.152 (+7.31%)		0.105	0.004
MG	103.357	>100	>4 (>30%)	0.172 (21.58%)		6.197	0.026
YUK	241.993	>100	2.934 (−5.13%)	0.145 (+2.19%)	0.135	0.093	0.004
sBM	106.846	22.617	3.685 (+19.16%)	0.165 (+16.35%)		0.714	0.021
PvS	55.513	5.257	3.073 (−0.61%)	0.137 (−3.23%)	0.028	0.039	0.002
DEM	113.037	>100	>4 (>30%)	0.114 (−19.23%)		2.345	0.014
MS-DEM	89.099	13.407	3.111 (+0.61%)	0.113 (−20.40%)	0.056	0.049	0.008
JS	240.452	>100	3.605 (+16.57%)	0.202 (+42.59%)		0.473	0.011

**Table 3:** Parameters of the 5-layer antireflection coating for matching free space and silicon as schematically illustrated in Figure 6a. *i* denotes the layer number. PEP (BTEP) represents the porous epoxy (BaTiO_3_–epoxy) composite.

Parameter	i=1	i=2	i=3	i=4	i=5
${\varepsilon^{\prime }}_{i}$	1.080	1.586	3.418	7.369	10.819
${\varepsilon^{\prime \prime }}_{i}$	0.003	0.030	0.135	0.514	0.920
Thickness (mm)	7.21	5.95	4.05	2.76	2.28
Type	PEP	PEP	BTEP	BTEP	BTEP
Volume fraction	93.2%	59.2%	1.9%	18.6%	28.0%

## Data Availability

Not applicable.
